# 791 Occupational Therapy in Operating Room An Emerging and Distinct Role in Collaborative Burn Care Practice

**DOI:** 10.1093/jbcr/irac012.341

**Published:** 2022-03-23

**Authors:** Allen R Espelita

**Affiliations:** University Medical Center, Las Vegas, Nevada

## Abstract

**Introduction:**

Numerous health professions face challenges and opportunities resulting in increasing contexts for service delivery. One element of this changing landscape for occupational therapy is the ongoing development and delivery of services in new or underdeveloped practice settings, often identified as emerging practice (Holmes & Scaffa, 2009). Often, occupational therapy practitioners work on interprofessional teams. Articulating occupational therapy's distinct value is necessary to communicate with clarity to those outside of the profession. According to the American Occupational Therapy Association (AOTA) Vision 2025, As an inclusive profession, occupational therapy maximizes health, well-being, and quality of life for all people, populations, and communities through effective solutions that facilitate participation in everyday living. One of the pillars identified in the AOTA 2025 vision is Collaboration: Occupational therapy excels in working with clients and systems to produce effective outcomes (AOTA, 2017).

**Methods:**

Through the initiative of one of the surgeons, she collaboratively integrated Occupational Therapy in the OR during the debridement and skin grafting process. While the patient is on sedation, the surgeon allowed OT to facilitate a range of motion, allowing optimal range of motion (ROM). The focus of the management in the acute phase of burn rehabilitation is the preservation of function through the prevention of deformities. Using a case study, the therapist's clinical and professional reasoning concerning a client's occupational performance (AOTA, 2020 p.20).

**Results:**

Occupational therapy specialists use theoretical principles and models, knowledge about the outcomes of disorders on participation, and existing evidence on the efficacy of interventions to guide their reasoning. Understanding this distinct role as an occupational therapist should start from the beginning of burn care management among patients with burns inside the operating room. Having said all this, our Rehabilitation Department Burn Therapy Team was allowed to learn the introductory course in scrubbing, gowning, and gloving for healthcare professionals.

**Conclusions:**

The idea of health care teams working together is not new. However, applying a culture of transformation, where concepts such as patient safety, mutual respect, shared decision making, and patient centered care are the norm, remains a novel and often fleeting idea in most burn care facilities (Sexton & Baessler, 2016).

